# Experience with the novel unifemoral parallel sheath technique in percutaneous intervention of chronic total coronary occlusions

**DOI:** 10.1186/s43044-021-00134-z

**Published:** 2021-02-05

**Authors:** Joerg Reifart, Konstanze Schilling, Christian W. Hamm, Nicolaus Reifart

**Affiliations:** 1 Department of Cardiology, Kerckhoff Heart and Thorax Center, Benekestr. 2-8, 61231 Bad Nauheim, Germany; 2 DZHK (German Center for Cardiovascular Research), partner site Rhein-Main, Frankfurt am Main, Germany; 3 Kliniken des Main-Taunus-Kreises GmbH, Hofheim, Germany; 4 Department of Cardiology, Petrus-Krankenhaus, Wuppertal, Germany

**Keywords:** Coronary artery disease, Chronic total occlusion, Access site, Percutaneous coronary intervention

## Abstract

**Background:**

Percutaneous interventions to address chronic coronary occlusions (CTO-PCI) often require simultaneous ipsi- and contralateral coronary injections. Although radial access is increasingly popular, bifemoral artery access is still the preferred choice of CTO operators.

The aim of this case series is to demonstrate the feasibility and safety of the unifemoral parallel sheath technique, which avoids two puncture sites, increases patient comfort, and improves procedure ergonomics.

It offers rapid second access to the femoral artery adjacent to the first sheath as well as closure by unilateral manual compression without or with 1 or 2 vascular closure devices.

**Results:**

We retrospectively evaluated the procedure results in 90 consecutive CTO patients where an ipsilateral parallel sheath access was considered. Placement of the second sheath uneventfully failed in two because of severe femoral calcification and narrowing. In 96.6%, the first sheath was 7 F (3.4% 6F), while the second sheath was 4 F in 22.7%, 5 F in 64.7%, and 6 or 7 F in 11.4% each.

No major complications nor severe bleeding events occurred, and the mean drop of hemoglobin was low (0.6 g/dL ± 0.86).

**Conclusion:**

In CTO-PCI requiring contralateral coronary injections or the retrograde technique, the ipsilateral parallel sheath technique might be a feasible alternative to the standard bifemoral or femoral-radial access.

## Background

Percutaneous coronary interventions of chronic total occlusions (CTO) are considered to be the most difficult of all percutaneous interventions.

In the last 15 years, several techniques and newly developed materials have helped to lift the success rates in experienced operators from about 50 to 90% [1]. Relevant contributions to success were the use of contralateral dye injection to visualize the anatomy of the occluded vessel, evolution of better imaging modalities, specific wires, microcatheters, the parallel wire technique, and the retrograde approach [2].

Ever since contralateral dye injection was first described in 1986, it has been done via a second sheath at a different access site [3].

With the increasingly popular transradial access for CTO-PCI, there are various access site combinations that might be used for contralateral opacification: bilateral femoral access, bilateral radial access, or femoral plus left or right radial access.

The novel unifemoral parallel sheath technique was first described in 2001, but reports of routine use in patients are missing.

A different method which does not require a second puncture site using only one guiding catheter for recanalization and for contralateral injections was described by Kiemeneij in 2014 and Yoshimachi in 2016. After coronary intubation and wiring, the operator must maintain wire or microcatheter position and turn the guide towards the contralateral vessel, a cumbersome technique that was not adopted since it appears unsuitable for most procedures [4, 5].

The parallel sheath technique initially was invented to lower the hurdle of contralateral injection during the course of a CTO-PCI (no need to prepare a second access site), to increase the patient’s comfort (only one compression bandage instead of two) and reduce the risk of access site-related complications which intuitively should be lower with 1 access site instead of 2 [6, 7].

In most individuals, the common femoral artery has a diameter of at least 7 mm so theoretically up to two 9 French sheaths (3 mm each) might be placed [8].

In this case series, we analyzed bleeding and access site complications as well as success rates of the parallel sheath technique performed by a single operator in 90 consecutive patients.

## Methods

From January 2008 until December 2019, a total number of 810 patients underwent CTO-PCI performed by a single highly experienced operator. In 598 cases, contralateral injections were required to visualize the vessel distal to the occlusion, and in 587/598, the parallel sheath technique was applied.

In our study, we are analyzing a subgroup of 90 consecutive patients in which laboratory controls were systematically scheduled the day after CTO-PCI.

All patients had a chronic coronary occlusion causing angina or dyspnea and proven viable myocardium assessed by echocardiography, magnetic resonance imaging, or scintigraphy.

Chronic total coronary occlusion was defined congruent with the EuroCTO club [9].

Procedural success was defined as TIMI flow 3 and a residual stenosis of 20% or less.

Major adverse cardiac events (MACE) were defined as new intra-hospital non-Q-wave and Q-wave myocardial infarction (defined as myocardial infarction by laboratory findings with or without development of new Q waves), recurrent angina requiring urgent repeat revascularization with PCI or coronary artery bypass grafting, stroke, death, pericardiocentesis, or surgical drainage of pericardial hematoma [10].

Minor bleeding was defined as any bleeding or reported large hematoma (with Hb drop < 3 g/dL) that led to a prolonged hospital stay for surveillance (BARC type 1 or 2) [11].

Major bleeding was defined as bleeding with hemoglobin drop of > 3 g/dL or requiring transfusion of any blood products (BARC 3a criteria or greater).

We assessed hemoglobin levels before and the day after the intervention as well as INR, thrombocytes, procedural time, and platelet inhibiting medication. Patients were allowed to eat and drink the day before as well as having a light breakfast the day of the procedure. To deal with potentially excessive dye consumption, intravenous saline was given the day before at a rate of 80 mL/h to all patients unless they had a severely reduced ejection fraction.

Patients were pre-loaded with acetylsalicylic acid 500 mg and Clopidogrel 600 mg or Ticagrelor 180 mg at the time of intervention unless they were already on dual antiplatelet therapy (DAPT). Oral anticoagulation (OAC) was stopped 24 h before the procedure.

All patients were considered to receive two unifemoral sheaths with one primary access for intervention and the second one for contralateral dye injection. If antegrade success was likely, 4 French sheaths were chosen. Planned or potential retrograde procedures required a sheath size of 5 French or greater.

**Fig. 1:**
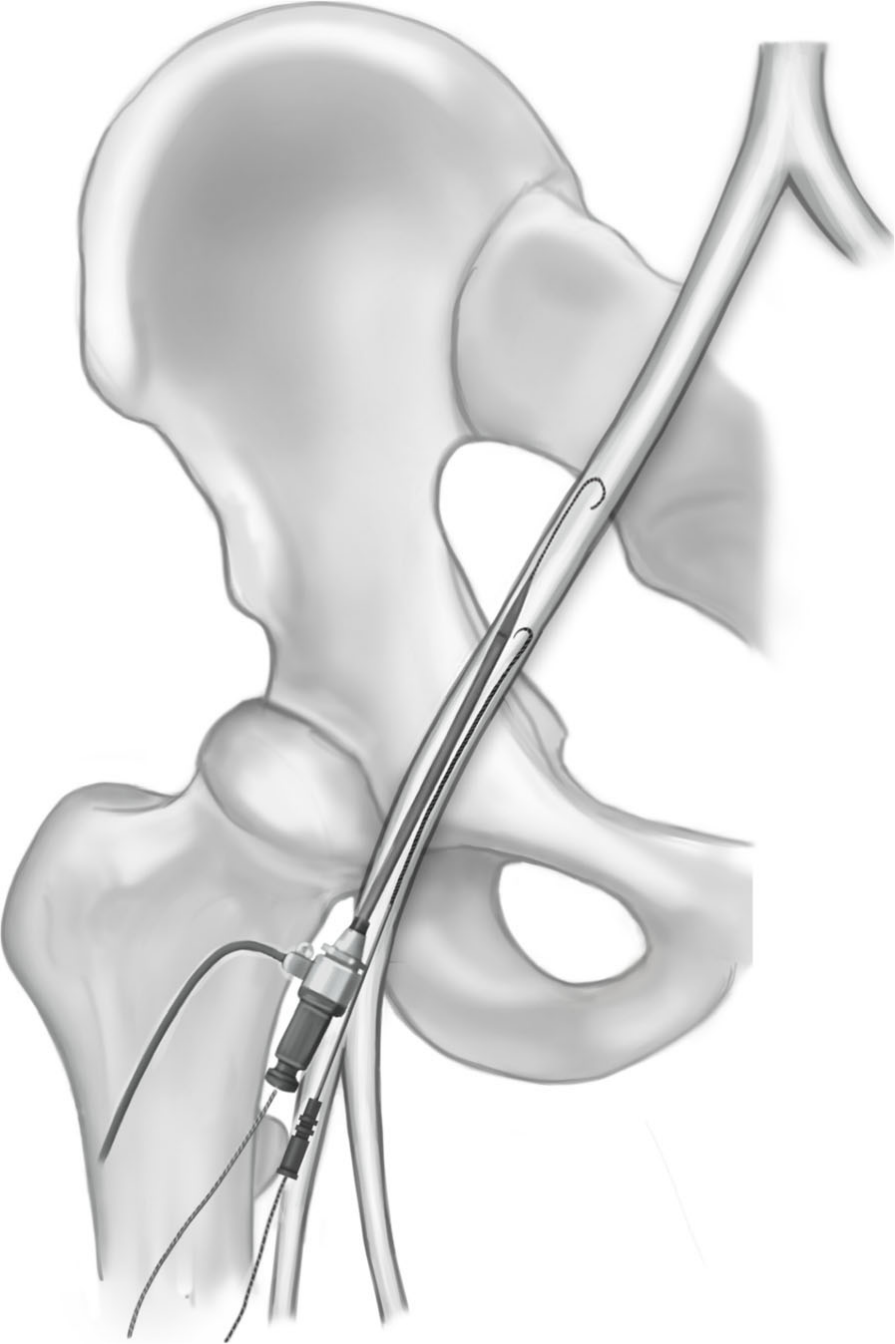
Initial puncture for the parallel sheath technique. The first sheath is used as a guide for the second puncture

**Fig. 2:**
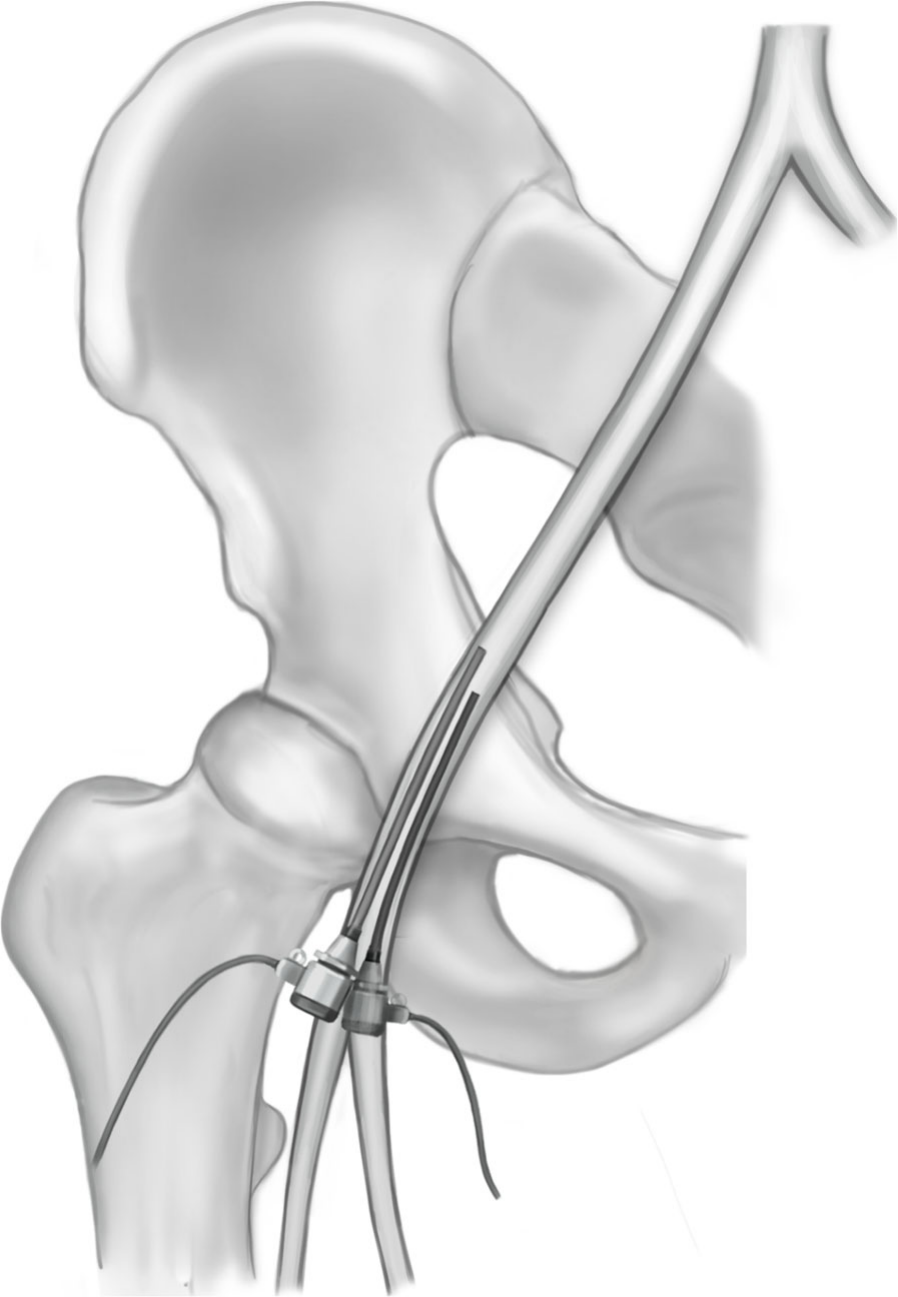
Introduction of the second sheath parallel to the first

**Fig. 3:**
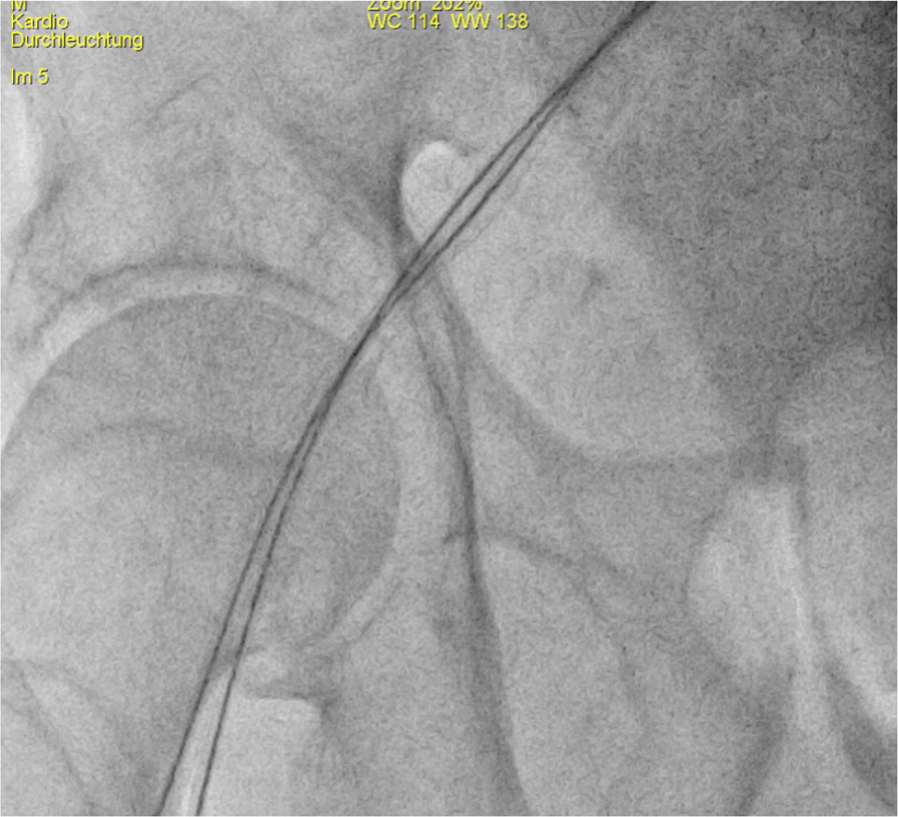
Fluoroscopically confirming the puncture site and straightening of femoral artery tortuosity with two wires in place

The larger size sheath was always put in first. The dilatator and Seldinger wire were left in place to help straighten the vessel and to guide the second puncture (Fig. 1). The smaller sheath was then placed medial to the first sheath unless there was not adequate space. The needle was guided strictly parallel and close to the first sheath making sure not to puncture it (Fig. 2). In some cases, the puncture and sheath placement were confirmed fluoroscopically (Fig. 3). In patients with severe femoral calcification and difficulty to insert the first sheath, the femoral artery was visualized with dye. In two cases, the operator deemed the vessel to be too narrow for a second sheath and the parallel sheath technique was abandoned (2.2%).

All procedures followed the same protocol with 10,000 IU of unfractionated heparin given as a bolus as soon as both sheaths were successfully placed. Roughly two thirds of the bolus were given through the sheath with the bigger diameter, the remaining third into the other sheath. Activated clotting time (ACT) was checked after 45 min while with BMI above 30 kg/m^2^, the ACT was checked sooner. Subsequent doses of unfractionated heparin were given according to the operator’s discretion with the goal to keep the ACT > 250 s during the procedure and < 250 s at its end. After the PCI was concluded, both sheaths were pulled immediately unless the ACT was still above 250 s, or the patient’s systolic blood pressure was above 180 mmHg. Reversal of heparin was not routinely performed.

**Fig. 4:**
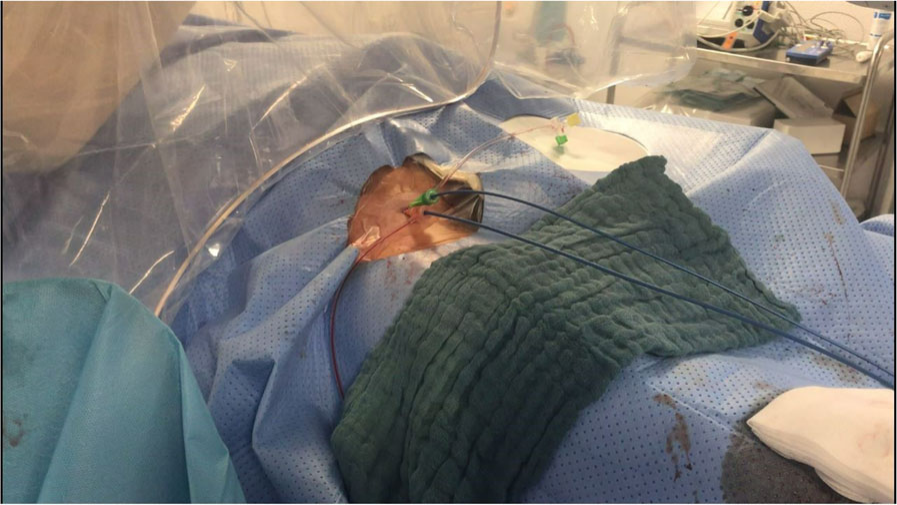
Dual access with guide catheters in place

**Fig. 5:**
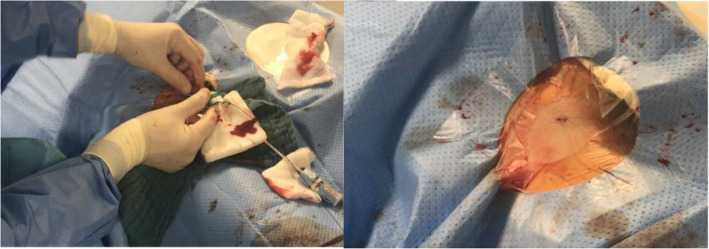
Optional closure with two vascular closure devices

No vascular closure devices were used in this case series, all sheaths were pulled and followed by manual compression only. More recently, two Angio-Seal vascular closure devices (Terumo Interventional Systems, Somerset, NJ) were used to close both puncture sites (if > 4 French) in most cases (Figs. 4 and 5).

Baseline data including procedural information, demographics, and comorbidities were collected prospectively in a dedicated database (ERCTO registry). Written consent was obtained. The case series was approved by the institutional research and ethics committee and complied with the Declaration of Helsinki.

Data analysis was done with the open access statistical software R 3.6.1 and R-Studio. All values are represented as mean with standard deviation.

## Results

The overall success rate of the CTO procedures was 86.6%.

Patient and procedural characteristics are shown in Table 1.

**Table 1:**
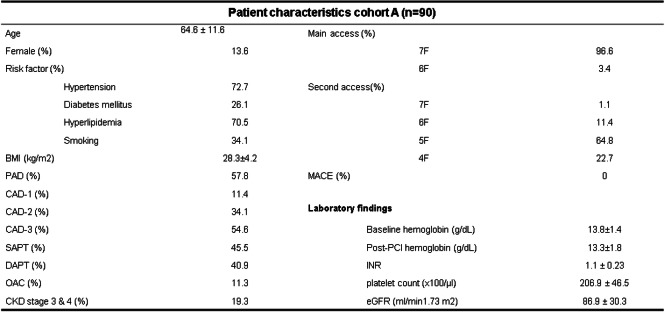
Patient characteristics of cohort A (*n* = 90)

Retrograde technique was used in 28.8% of cases—most which had previously failed an antegrade recanalization attempt.

In 96.6%, the primary sheath size was 7 French, while the second sheath was greater than 4 French in 77%, allowing retrograde procedure.

97.7% of the prospective cases were successfully performed with two sheaths in a single artery.

There were no major or minor bleeding complications nor major adverse cardiovascular events.

The difference between pre- and post-PCI hemoglobin was 0.6 g/dL (± 0.86) (Table 1).

## Discussion

Most CTO-PCI cases require dual arterial access which is likely to increase the rate of access site complications, although this was not confirmed by a large registry [12].

Vascular complications following coronary interventions may vary between 11.3%, 9.4%, and 2.1% for transfemoral and 3.5%, 3.4%, and 0.9% following transradial approach [13–15].

The unifemoral parallel sheath technique, first described in 2001, has several advantages over bifemoral access: For one, the second access is more rapidly attained than contralateral femoral puncture. Additionally, there is increased patient comfort, and finally the technique was shown to effectively overcome severe kinking of the iliac artery [7]. However, it remained unclear if two sheaths inserted into the same femoral artery would increase access site complications.

In the observation presented here, the parallel sheath technique was feasible and safe in most cases.

The observed drop in hemoglobin of 0.6 g/dL (a greater part of which might be attributed to backbleeding and blood testing), compares well with the previously reported 0.8 g/dL drop with a single access [15].

In the literature, access site-related complications are reported to occur in about 2% with femoral/femoral access, 1.2% following femoral/radial approach, and 0.9% with radial/radial access [14].

Although radial/radial was reported to have the lowest complication rate, it is not ideal for complex cases (e.g., when parallel wires or IVUS are required) and is hampered with longer fluoroscopy and procedure time [16].

The unifemoral dual sheath technique represents a reliable alternative to dual arterial access that will increase the readiness to upgrade from single to dual sheaths using the first sheath as a track and not needing to prepare a second access site. In addition, it eases guide catheter manipulation in cases with severe tortuosity of the iliac artery [17].

Since the right femoral access site was shown to bring about the lowest radiation exposure, one might speculate that the novel technique is likely to be advantageous in this regard as well [18, 19].

### Limitations

Registry patients are not monitored as rigorously as in randomized controlled studies. Since our patients were discharged the day after the procedure, we cannot exclude that late bleedings may have occurred, although it would have been very likely that upon bleeding the patients would have been sent back to our hospital. Apart from the retrospective design, the study is lacking a control group that allows comparison to an alternative access. As a single operator study, the results may not be generalized and operators with less experience may need to perform the puncturing with assistance of ultrasound [20].

## Conclusion

The parallel sheath technique with unifemoral dual access appears an easy and safe alternative to approaches with two access sites with complication rates similar to those of a single transfemoral sheath.

## Data Availability

The data presented in this article can be made shared upon request.

## Abbreviations

ACT: Activated clotting time
CAD: Coronary artery disease
CKD: Chronic kidney disease
CTO: Chronic coronary occlusions
DAPT: Dual antiplatelet therapy
MACE: Major adverse cardiac events
MRI: Magnetic resonance imaging
OAC: Oral anticoagulation
PAD: Peripheral artery disease
PCI: Percutaneous coronary intervention
SAPT: Single antiplatelet therapy

## References

[1] Konstantinidis NV (2018). Temporal trends in chronic total occlusion interventions in Europe. Circulation.

[2] Weisz G, Moses JW (2010). Contemporary principles of coronary chronic total occlusion recanalization. Catheter Cardiovasc Interv.

[3] Grollier G (1986). Transluminal dilatation of occluded coronary arteries. Value of retrograde opacification of the occluded vessel by the contralateral artery. Archives des maladies du coeur et des vaisseaux.

[4] Yoshimachi F, Torii S, Naito T (2016). A novel percutaneous coronary intervention technique for chronic total occlusion: contralateral angiography with a single guiding catheter. Catheter Cardiovasc Interv.

[5] Kiemeneij F (2014). Simultaneous transradial coronary angioplasty and contralateral coronary angiography with a single guide catheter for total coronary occlusions. J Invasive Cardiol.

[6] N., R (2001). Contralateral injections for chronic total occlusions using 4 F and the same groin. in *Chronic total occlusion pathophysiology, intervention and expert case management* (ed. Katoh O, M.J., Reifart N, Virmani R).

[7] Nicolaus R, Navid S (2014). The parallel sheath technique in severe iliac tortuosity: a simple and novel technique to improve catheter manoeuvrability. EuroIntervention.

[8] Sandgren T, Sonesson B, Ahlgren ÅR, Länne T (1999). The diameter of the common femoral artery in healthy human: influence of sex, age, and body size. J Vasc Surg.

[9] Di Mario C (2007). European perspective in the recanalisation of chronic total occlusions (CTO): consensus document from the EuroCTO Club. Eurointervention.

[10] Galassi AR (2015). Retrograde recanalization of chronic total occlusions in Europe: procedural, in-hospital, and long-term outcomes from the multicenter ERCTO registry. J Am Coll Cardiol.

[11] Mehran R (2011). Standardized bleeding definitions for cardiovascular clinical trials: a consensus report from the Bleeding Academic Research Consortium. Circulation.

[12] Lee KH (2010). Periprocedural hemoglobin drop and contrast-induced nephropathy in percutaneous coronary intervention patients. Korean circulation journal.

[13] Rathore S (2009). A comparison of the transradial and the transfemoral approach in chronic total occlusion percutaneous coronary intervention. Catheteriz Cardiovasc Intervent.

[14] Poletti E et al (2019) Conventional vascular access site approach versus fully trans-wrist approach for chronic total occlusion percutaneous coronary intervention: a multicenter registry. Catheteriz Cardiovasc Intervent 96(1):E45–E52 10.1002/ccd.28513 31596537

[15] Kinnaird T (2017). Vascular access site and outcomes among 26,807 chronic total coronary occlusion angioplasty cases from the British Cardiovascular Interventions Society National Database. JACC: Cardiovascular Interventions.

[16] Alaswad K (2015). Transradial approach for coronary chronic total occlusion interventions: insights from a contemporary multicenter registry. Catheteriz Cardiovasc Intervent.

[17] Reifart N, Sotoudeh N (2014). The parallel sheath technique in severe iliac tortuosity: a simple and novel technique to improve catheter manoeuvrability. Eurointervention.

[18] Shah B (2013). Radiation exposure during coronary angiography via transradial or transfemoral approaches when performed by experienced operators. American heart journal.

[19] Kallinikou Z (2016). Radiation exposure of the operator during coronary interventions (from the RADIO Study). The American journal of cardiology.

[20] Dakhil B (2017). Is ultrasound guidance contributive to vascular access in endovascular therapy?. J Med Vasc.

